# SAKK 21/12: a phase II trial of transdermal CR1447 in breast cancer patients

**DOI:** 10.1530/EO-21-0009

**Published:** 2022-02-10

**Authors:** Marcus Vetter, Karin M Rothgiesser, Qiyu Li, Hanne Hawle, Wolfgang Schönfeld, Karin Ribi, Salome Riniker, Roger von Moos, Andreas Trojan, Elena Kralidis, Mathias Fehr, Andreas Müller, Beat Thürlimann, the Swiss Group for Clinical Cancer Research (SAKK)

**Affiliations:** 1 Medical Oncology, University Hospital Basel, Basel, Switzerland; 2 Medical Oncology, Hematology and Immunotherapy, Cantonal Hospital Baselland, Medical University Clinic, Liestal, Switzerland; 3 Swiss Group for Clinical Cancer Research (SAKK) Coordinating Center, Bern, Switzerland; 4 CURADIS GmbH, Erlangen, Germany; 5 IBCSG, International Breast Cancer Study Group, Bern, Switzerland; 6 Breast Cancer Center, Cantonal Hospital St. Gallen, St. Gallen, Switzerland; 7 Medical Oncology, Cantonal Hospital Chur, Chur, Switzerland; 8 Medical Oncology, Hirslanden Klinik Im Park, Zurich, Switzerland; 9 Medical Oncology, Cantonal Hospital Aarau, Aarau, Switzerland; 10 Medical Oncology, Hospital Thurgau, Thurgau, Switzerland; 11 Medical Oncology, Cantonal Hospital Winterthur, Winterthur, Switzerland

**Keywords:** metastatic breast cancer, estrogen receptor, androgen receptor, CR1447, endocrine therapy

## Abstract

**Objective:**

CR1447, a novel transdermal formulation of 4-hydroxytestosterone, has aromatase-inhibiting and androgen receptor (AR)-modulating properties (IC_50_4.4 nM) with antitumor effects against AR-positive tumor cells *in vitro.* This trial investigated the efficacy and safety of CR1447 for patients with metastatic estrogen receptor-positive (A) and AR-positive triple-negative breast cancers (B).

**Design and methods:**

(A) included patients with at most one prior endocrine therapy line without progression ≥6 months, whereas (B) included patients with ≤2 prior chemotherapy lines, all displaying advanced signs of disease. The primary endpoint was disease control at week 24 (DC24). The null hypothesis was DC24 ≤30% (A) and ≤15% (B). Thirty-seven patients were recruited (29 in (A) and 8 in (B)); accrual was stopped following an interim analysis demonstrating futility in (A) and slow accrual in (B).

**Results:**

DC24 was attained in 5/21 (95% CI: 8.2–47.2) patients in (A) and none in (B). The median progression-free survival was 5.1 months (95% CI: 2.5–5.6) in (A) and 2.5 months (95% CI: 0.7–2.6) in (B). The median overall survival was 24.6 months (95% CI: 22.9–not applicable) in (A) and 10.8 months (95% CI: 3.3–10.9) in (B). CR1447 had a favorable safety profile without treatment-related grade 3–5 toxicities in (A). Especially no side effects linked to androgenic effects were observed.

**Conclusions:**

Despite this trial being negative, the 24% DC24 rate in a second-line setting, and the prolonged partial response experienced by a patient, indicate activity. Further evaluation of CR1447 in endocrine-sensitive patients or combination trials appears warranted.

## Introduction

Estrogen receptor-positive (ER+) breast cancer is the most common subtype of breast cancer, and generally, has a good prognosis (Onitilo *et al.* 2009). Despite new treatment options, a relatively high proportion of patients develop metastatic relapse, which is estimated to occur in 20–30% of patients (Pan *et al.* 2017). Approximately, 70–95% of ER+/HER2− breast cancers do also express the androgen receptor (AR) (Chen *et al.* 2020).

For metastatic ER+/HER2− breast cancer without visceral crisis (which would be indicated by severe organ dysfunction assessed by signs, symptoms or laboratory studies, resulting from the rapid progression of cancer), endocrine therapy with aromatase inhibitors (AIs), (such as letrozole), tamoxifen, or fulvestrant, is the therapeutic cornerstone (Palmieri *et al.* 2014). Recently, a combination of endocrine therapy and cyclin-dependent kinase 4/6 (CDK4/6) inhibitors was successfully applied clinically (Turner *et al.* 2015, Hortobagyi *et al.* 2016, Sledge *et al.* 2017), resulting in an extended progression-free survival (PFS), alongside survival benefits in endocrine-sensitive patients when combined with fulvestrant or an AI (Turner *et al.* 2018, Im *et al.* 2019). These combinations are now the standard of care in metastatic ER+/HER2− breast cancer in first, second, and later-line settings (Dickler *et al.* 2017, Telli*et al.* 2019). Taking into consideration that CDK4/6 inhibitors usually lead to drug resistance, new treatment combinations are warranted (Turner *et al.* 2017).

Concerning testosterone, this agent was already employed in the 1930s. Tumor response was observed with the use of testosterone, especially in patients with the bone-only disease (Schwander & Marvin 1947). However, several side effects, such as virilization, were also observed (Segaloff *et al.* 1964). In brief, 4-hydroxytestosterone (4-OHT (CR1447)) is a steroidal molecule with two different mechanisms of action, acting as a steroidal AI and binding to the AR with high affinity (IC_50_: 4.4 mM) (Zweifel *et al.* 2017). The binding to the receptor is higher than testosterone itself. 4-OHT does not exert any androgenic properties in a relevant animal model (Hershberger assay) but maintains anabolic properties (data not published). Therefore, 4-OHT is best characterized as ‘selective androgen receptor modulator’ (SARM). 4-OHT exhibits potent antitumor activity against AR-positive ER+ and ER− tumor cell lines (e.g. T47D, ZR-75, MDA-MB453) *in vitro* (unpublished data). *4-OHT* and *4-OHA*(4-hydroxyandrost endione, a strong aromatase inhibitor, form a redox system and metabolites of both of them can be detected in blood and urine (Johannessen *et al.* 1993, Kohler *et al.* 2007). Moreover, the combination of 4-OHT and the CDK4/6 inhibitor palbociclib or chemotherapeutic agent (docetaxel) demonstrates synergistic activity in cell cultures (unpublished first preclinical data).

Previous phase I data on CR1447 demonstrated a certain degree of efficacy and favorable toxicity profile, without any drug-related dose-limiting toxicities (Zweifel *et al.* 2017). The most common adverse events (AEs) were elevated triglyceride and liver enzyme levels, as well as anemia, with a recommended phase II dose of 400 mg/day given continuously. In the phase I study, all AEs were reversible and did not exceed Common Terminology Criteria for Adverse Events (CTCAE) Grade I–II.

Pharmacokinetic analysis indicated a dose-dependent increase in serum levels of 4-OHT reaching about 6 ng/mL at a single topically administered dose of 400 mg; 4-OHA levels were mostly below the detection limit. The data indicate sufficient uptake of 4-OHT into the systemic blood circulation and high stability of 4-OHT (Zweifel *et al.* 2017).

Owing to the function of CR1447 as an AI and AR-modulator, patients with metastatic or locally advanced ER+/HER2− including also ER+/HER2− AR+ breast cancer and triple-negative breast cancers (TNBC) AR+ breast cancer were considered suitable candidates for a phase II trial.

## Patients and methods

This multicenter phase II stratified trial SAKK 21/12 (NCT No. 02067741) recruited patients into two cohorts*,*namely those with (ER+)/HER2− disease into Cohort A (A), and those with AR+ TNBC (ER− (<1%), progesterone receptor (PgR)− (<1%), HER2−) metastatic breast cancer into Cohort B (B). The trial was approved by the central and local ethics committees and the Swiss Agency for Therapeutic Products (Swissmedic). This Trial complies with the Declaration of Helsinki and the subjects gave their informed written consent.

Postmenopausal patients with either ER+/HER2− or AR+ TNBC, who needed systemic therapy, were enrolled into (A) and (B), respectively. The histologic definitions were ER+ (≥1%) or PgR+ (≥1%) and HER2− for (A) patients and AR+ (> 0%) and TNBC for (B). In (A), the patients were initially required to have undergone ≥1 and ≤3 lines of endocrine treatments (including adjuvant treatments). Later on, these inclusion criteria were changed to only one line of prior endocrine therapy administered for at least 6 months, without any previous chemotherapy. In (B), eligible patients were required to have undergone ≤2 chemotherapy lines for metastatic disease, with AR+ (>0%) determined by the central pathology on the most recent tumor materials.

CR1447 was supplied in aluminum-coated stick packs as a 4 g ointment containing 2.5% of active 4-OHT. CR1447 was produced under good manufacturing practice (GMP) conditions and distributed by Promedipharm GmbH (Germany). CURADIS Pharma was involved in the development of this drug. A 400 mg dose of CR1447 dose was applied daily to haunches and thighs (200 mg in the morning and evening, respectively). The patients were advised to use one pack of ointment for each thigh, and the treatment was continued until disease progression or until unacceptable toxicity was reported.

To control treatment compliance, all patients received a diary to keep a record of how CR1447 was administered; compliance followed-up using each patient’s diary. A clinical assessment was performed at baseline and the beginning of every treatment cycle (every 21 days), including a physical examination as well as World Health Organization performance status, weight, blood pressure, and heart rate. Hematologic values, renal function, hepatic functions, and triglyceride levels were assessed on day 1 of each cycle. The patient-reported outcomes and quality of life (QoL) were based on the Functional Assessment of Cancer Therapy-Endocrine Subscale (FACT-ES) questionnaire on day 1 of weeks 4 and 24 and at trial treatment discontinuation.

For tumor assessment, ≥1 measurable lesion according to RECIST 1.1 was required. A tumor assessment using CT and bone scans was mandatory at baseline, week 12, and week 24. After 24 weeks, the tumor assessment could be performed after a minimum of 6 months. Confirmation of a response for patients with a partial response (PR) and complete response (CR) according to RECIST 1.1 was mandatory at least 4 weeks after the first PR or CR. The CT images documenting CR, PR, and stable disease (SD) were available in a digitalized form (Digital Imaging and Communications in Medicine format) for an independent response review.

The patient-reported endocrine symptoms were assessed by the 19-item Functional Assessment of Cancer Therapy – Endocrine Subscale (FACT-ES) (Fallowfield *et al.* 1999) at baseline, week 4, week 24, and treatment discontinuation (whichever occurred first). The response options ranged from 0 (‘not at all’) to 4 (‘very much a problem’). Individual item scores were transformed so that a higher total score indicates a better QoL. To gain clinically useful information regarding undesirable effects, we categorized the patient responses to each FACT-ES question. Scores of three (‘quite a bit’) and four (‘very much’) were considered ‘clinically relevant’. Scores of 0, 1, or 2 (‘not at all’, ‘a little bit’, and ‘somewhat’ a problem, respectively, were considered ‘not clinically relevant’ (Fallowfield *et al.* 2004).

To assess the feasibility of the CR1447 application, we adapted a questionnaire previously used in cancer patients for skincare management (Haley *et al.* 2011). The patients were invited to indicate their skin type and rate the CR1447 tolerability, its absorption, and their overall experience with it. To assess the burden of application, an adapted version of the indicator for coping effort was used (‘How much effort does it cost you to use the ointment as prescribed?’; 0 = ‘no effort at all’; 6 = ‘a great deal of effort’) (Hürny *et al.* 1993).

### Statistical design and analyses

The primary endpoint was disease control at week 24 (DC24). Overall, 45 patients in (A) were needed to demonstrate the DC24 rate to be at least 30% under the assumption that DC24 (rDC24) was 50% with a one-sided 5% Type I error and 80% power using Simon’s two-stage optimum design. For (B), 37 patients were needed to demonstrate rDC24 to be at least 15% under the assumption that rDC24 was 30% with a 10% one-sided type I error and 80% power using Simon’s two-stage minimax design modified using Herndon’s approach. Interim analyses were planned to be performed as soon as the DC24 of the first 16 patients in (A) and 18 patients in (B) were available.

The secondary endpoints included disease control rate at week 12 (DC12), change in tumor size at 12 weeks (CTS12), PFS, PFS at 24 weeks (PFS24), overall survival (OS), overall response (OR), AEs according to CTCAE v4.0, patient-reported outcomes (endocrine symptoms and feasibility of CR1447 application), and translational research endpoints (not reported here).

DC24 was based on patients achieving CR or PR within the first 26 (24 + 2) weeks or SD for at least 22 (24−2) weeks. Patients with only SD and those without tumor assessment between 22 and 26 weeks could be considered a DC24 success only if a subsequent assessment after 26 weeks showed SD. DC12 was defined similarly with a 12 ± 1 week time window. CTS12 was the percentage change from baseline to week 12 ± 1 in the sum of target lesion diameters. PFS was the time from registration until progression or death, whichever occurred first, and OS was the time from registration until death. OS was defined as CR or PR during treatment. For patient-reported outcomes, changes in FACT-ES total scores were calculated by subtracting the baseline score from the score at each time point. A negative change indicated worsening of patient-reported endocrine symptoms. All time-to-event endpoints were estimated using the Kaplan–Meier method and are expressed as median and their 95% CI. All binary endpoints are expressed as percentages and their exact 95% CI. The analysis was performed using the SAS 9.4 and R 3.5.0 software.

## Results

**Figure 1:**
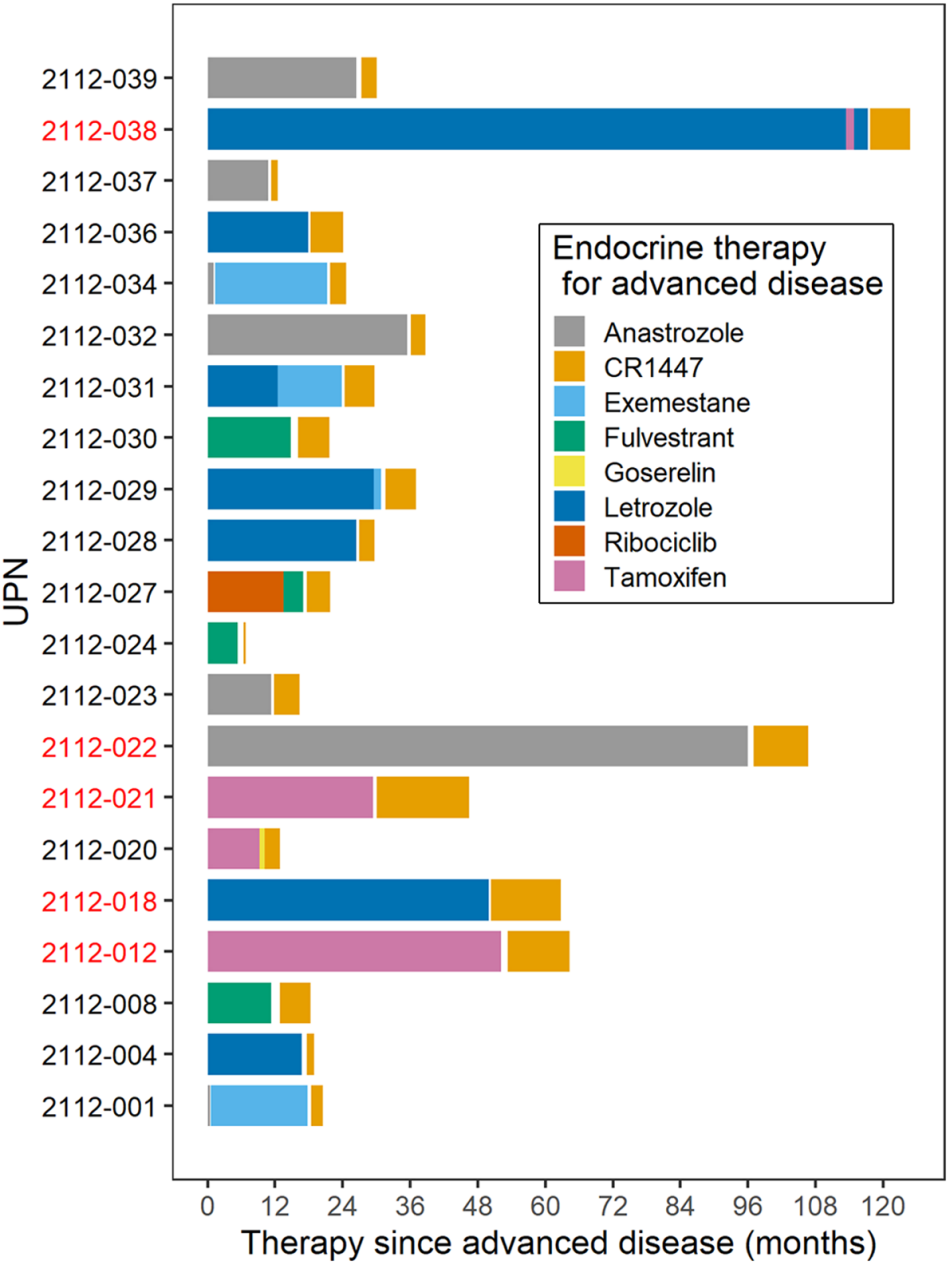
Prior endocrine therapy cohort. Patients who reached DC24 had longer previous endocrine therapy than patients without DC24.

Between June 2016 and February 2018, 29 and 8 patients were recruited into (A) and (B), respectively. Accrual for (A) was stopped early due to futility demonstrated in the interim analysis. Eight patients were not eligible after an amendment clarifying the eligibility criteria, because they had >1 prior chemotherapy line. Twenty-one eligible patients were analyzed for efficacy. Accrual for (B) was likewise stopped early owing to slow accrual, with eight patients being analyzed for efficacy. All patients in (A) and (B) were considered for the safety analysis (Fig. 1). The patients’ characteristics are presented in Table 1. Overall, 223 treatment cycles ((A) 199 cycles, (B) 24 cycles) of CR1447 were administered. Until the data cut-off, two patients in (A) were still on treatment. In (A), the median treatment duration was 12.6 weeks, and it was 9 weeks in (B). The total dose given was 34,800 mg (range 4400–199,900) in (A) and 24,500 mg (range: 3800–8500) in (B).

**Table 1:** Baseline characteristics of all patients by stratum – categorical variables.

Variable	A (*N* = 29)	B (*N* = 8)
	*n* (%)	*n* (%)
WHO performance status		
0	16 (55.2)	4 (50.0)
1	13 (44.8)	4 (50.0)
ER status		
Negative	0 (0.0)	8 (100.0)
Positive	29 (100.0)	0 (0.0)
PgR status		
Negative	8 (27.6)	8 (100.0)
Positive	21 (72.4)	0 (0.0)
AR status		
Negative	2 (6.9)	0 (0.0)
Positive	27 (93.1)	8 (100.0)
Measurable disease		
No	7 (24.1)	1 (12.5)
Yes	22 (75.9)	7 (87.5)
Locally advanced tumor		
No	29 (100.0)	8 (100.0)
Location of metastases without primary tumor (more than one applicable)		
Bone	22 (75.9)	5 (62.5)
Brain	0 (0)	2 (25.0)
Left breast	2 (6.9)	1 (12.5)
Liver	11 (37.9)	2 (25.0)
Lung	11 (37.9)	7 (87.5)
Lymph node	10 (34.5)	5 (62.5)
Right breast	0 (0.0)	0 (0.0)
Skin	2 (6.9)	1 (12.5)
Other	6 (20.7)	2 (25.0)

AR, androgen receptor; ER, estrogen receptor; PgR, progesterone receptor; WHO, World Health Organization.

### Primary and secondary efficacy endpoints

Of the 21 eligible patients in (A), 5 (23.8%, 95% CI: 8.2–47.2%) reached DC24, the objective set for (A) being a DC24 rate >30%, which was not confirmed. The result for (A) proved negative.

Of the eight eligible patients in (B), no patient (0%, 95% CI: 0.0–36.9%) reached DC24, the objective set for (B) being a DC24 rate >15%, which was not confirmed. The result for (B) proved negative as well.

We also investigated the association between DC24 and the length of previous endocrine therapy in eligible patients in (A). The duration before endocrine therapy was longer in patients who attained DC24 than in those who did not (Fig. 1). A closer investigation of these five patients would be required to understand what made them ‘endocrine sensitive’ in relation to the AR intervention.

### Secondary endpoints

Of the 21 eligible patients in (A), 13 (61.9%) reached DC12 (95% CI: 38.4–81.9%), whereas among the 8 eligible patients in (B), 0 (0%) reached DC12 (95% CI: 0.0–36.9%). A subgroup analysis of (A) demonstrated an insignificant DC12 difference between positive and negative PgR (60.0%, 95% CI: 22.3–95.7% vs 66.7%, 95% CI: 32.3–83.7%, respectively).

#### Cts12

Considering the CTS12 assessment in (A) and (B), nine and two patients could be evaluated, respectively. According to the inclusion criteria, all patients had ≥1 measurable/evaluable lesion according to RECIST. Eight patients had no target lesions at baseline. Eight patients had no tumor assessment at week 12 because they stopped treatment before week 12 and two patients had no tumor assessment at week 12 (±1 week) The median tumor size change was +3.6% for (A) and +29.6% for (B). For (A), the week 12 changes were in the range of −41.9 to +42.5%. We observed the descriptive difference in CTS12 between patients with positive and negative PgR (+2.3% vs +5.2%, respectively). An increase in tumor size was observed in most patients. A reduction in tumor size was observed in only two patients.

#### PFS

**Figure 2:**
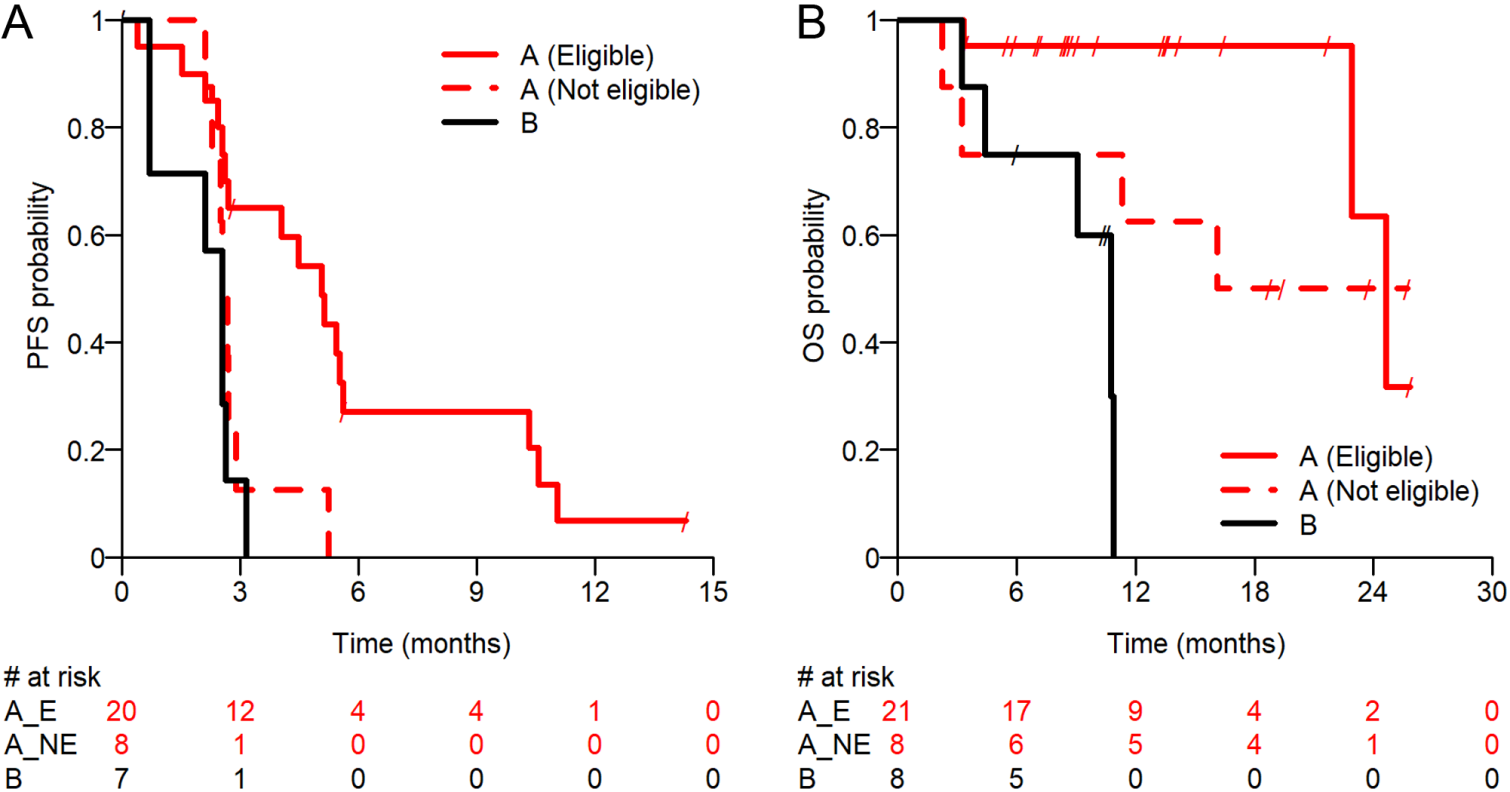
Outcome of patients in cohort A and B. PFS and OS probability. A_E, stratum A eligible patients; A_NE, stratum A not eligible patients; OS, overall survival; PFS, progression-free survival.

The number of eligible patients in (A) and (B) who experienced RECIST progression or died were 7 and 17, respectively. The median PFS in the eligible patients was 5.1 months (95% CI: 2.5–5.6) in (A) and 2.5 months (95% CI: 0.7–2.6) in (B). The Kaplan–Meier graphs for PFS are shown in Fig. 2A. A descriptive subgroup analysis involving eligible patients with either PgR− or PgR+ tumors revealed a small between-group difference in PFS (5.1 months vs 4.5 months) for (A). The PFS24 was 32.5% (95% CI: 13.5–53.3) for (A). Given that (B) patients and non-eligible (A) patients experienced events before 24 weeks, their PFS24 was 0%. A descriptive subgroup analysis involving PgR− and PgR+ tumor patients in (A) revealed similar results in PFS24 (33.3% vs 32.1%).

#### OS

The median OS was 24.6 months (95% CI: 22.9–not applicable (NA)) in (A) and 10.8 months (95% CI: 3.3–10.9) in (B). A subgroup analysis involving PgR− and PgR+ tumors patients in (A) showed a median OS of 22.9 months (95% CI: 3.4–22.9) and NA, respectively. The Kaplan–Meier graphs for OS are shown in Fig. 2B.

#### OR

Only one patient with ER+ and PgR+ tumor reached partial remission. The eligible patients’ OR was 4.8% (95% CI: 0.1–23.8%) in (A), whereas it was 0.0% in (B).

#### Patient-reported outcomes

Overall, no changes in endocrine symptoms (i.e. FACT-ES total score) were observed. For (A), the median values remained roughly the same, whereas the median symptom scores decreased (worsened) from the baseline to week 4. The proportion of patients who reported a symptom as clinically relevant varied according to the symptoms, but did not increase within 4 weeks of treatment, except for hot flushes. More than half (56%) of patients were tolerating CR1447 as well as or even better than other products after 4 weeks of treatment. Most patients rated the overall experience as ‘ok’, ‘good’, or ‘very good’ (83%). However, 53% of patients indicated that they would have preferred the treatment to be administered as tablets rather than ointment.

#### Toxicity and AEs

The treatment-related AEs and their relation to the treatment in (A) and (B) are listed in Table 2. Generally, CR1447 was well-tolerated. All patients experienced at least one AE, the majority of which were considered unrelated to treatment. The most common treatment-related AEs in (A) were of grade 1 or 2, including dry skin (12), nausea (5), fatigue (4), and maculopapular rash (4). In (B), four patients experienced treatment-related dry skin of grade 1 or 2. During or within the 30 days following the start of the treatment, two patients died due to disease progression. Both of the deaths were deemed unrelated to trial treatment.

**Table 2:** Summary of treatment-related AEs by AE type and highest grade for patients stratified by stratum.

	Term	A (*N*^a^ = 23)		B (*N*^a^ = 7)		
		G1	G2	G1	G2	G3
Gastrointestinal disorders	Abdominal pain	2 (8.7%)				
	Constipation	1 (4.3%)	1 (4.3%)	1 (14.3%)		
	Diarrhea	2 (8.7%)				
	Dry mouth	1 (4.3%)				
	Gastrointestinal disorders - dry lips	1 (4.3%)				
	Nausea	4 (17.4%)	1 (4.3%)	1 (14.3%)		
	Stomach pain	2 (8.7%)				
	Vomiting	1 (4.3%)				
General disorders and administration site conditions	Chills	1 (4.3%)				
	Fatigue	2 (8.7%)	2 (8.7%)		1 (14.3%)	1 (14.3%)
	Flu-like symptoms	2 (8.7%)				
	General disorders and administration site conditions – Mucosa dryness	1 (4.3%)				
Investigations	Investigations – Alanine aminotransferase increased and Aspartate aminotransferase increased		1 (4.3%)			
Metabolism and nutrition disorders	Anorexia	2 (8.7%)				
	Hypertriglyceridemia		1 (4.3%)			
Musculoskeletal and connective tissue disorders	Arthralgia	1 (4.3%)		1 (14.3%)		
	Bone pain		1 (4.3%)			
	Myalgia		1 (4.3%)			
	Pain in extremity	2 (8.7%)				
Nervous system disorders	Facial nerve disorder		1 (4.3%)			
	Headache	1 (4.3%)				
Psychiatric disorders	Agitation	2 (8.7%)	2 (8.7%)			
	Depression		1 (4.3%)	1 (14.3%)		
	Insomnia		1 (4.3%)			
	Psychiatric disorders – emotionally fragile			1 (14.3%)		
Reproductive system and breast disorders	Breast pain	3 (13.0%)				
	Vaginal dryness	2 (8.7%)				
Skin and subcutaneous tissue disorders	Alopecia	1 (4.3%)				
	Dry skin	10 (43.5%)	2 (8.7%)	3 (42.9%)	1 (14.3%)	
	Pruritus	1 (4.3%)	2 (8.7%)	1 (14.3%)		
	Rash maculopapular	2 (8.7%)	2 (8.7%)	1 (14.3%)		1 (14.3%)
	Skin and subcutaneous tissue disorders – Pimple inguinal	1 (4.3%)				
	Skin atrophy		1 (4.3%)			
Vascular disorders	Hot flashes	2 (8.7%)				

^a^Number of patients who had treatment-related AEs.

AE, adverse event.

#### Laboratory values

During the treatment, the most common abnormal laboratory values (grade 1) for (A) and (B) were hypertriglyceridemia (66.7 and 42.9%, respectively), increased aspartate aminotransferase (41.7 and 57.1%, respectively), increased creatinine (41.7 and 14.3%, respectively), and anemia (29.2 and 4.2%, respectively). A listing of the most common abnormal laboratory values reported in (A) and (B) is provided in Table 2.

## Discussion

There is a high unmet medical need for new systemic modalities to treat metastatic breast cancer, particularly the aggressive TNBC subtype (Hudis & Gianni 2011). In ER+/HER2− breast cancer, CDK4/6, mammalian targets of rapamycin inhibitors, and phosphoinositide 3-kinase inhibitors have substantially improved breast cancer prognosis (Janku *et al.* 2012, Piccart *et al.* 2014, Hamilton & Infante 2016). However, drug resistance is still a major issue that results in treatment failure (Ali & Coombes 2002, Lange & Yee 2011, Oesterreich & Davidson 2013, Robinson *et al.* 2013, Turner *et al.* 2017).

CR1447 comprises a novel therapeutic principle (4-OHT, an AR modulator) in a new formulation for topical endocrine therapy, with proven transdermal delivery and good tolerance in a previous phase I trial. Only moderate undesirable effects were observed (Zweifel *et al.* 2017), with the most common being elevated triglyceride levels and abnormal liver function tests.

A study on patients who underwent heavy pretreatment for ER+ metastatic breast cancer re-examined the role of testosterone (Boni *et al.* 2014). The analyzed data revealed a clinical benefit rate exceeding 50%, with occasional instances of virilization and dysphonia being reported. The authors concluded their research by stating that testosterone should be further evaluated in hormone-sensitive metastatic breast cancer patients.

In our study, CR1447 failed to reach its primary DC24 endpoint in both cohorts and is therefore a negative trial. In (A), however, CR1447 showed efficacy signs, with a clinical benefit rate of 61.9% at DC12 and 23.8% at DC24 in eligible patients (endocrine-sensitive population, at least 6 months duration of prior endocrine treatment). On the contrary, the non-eligible, non-endocrine-sensitive population did not respond to CR1447. The chosen endpoint of 30% DC24 could be deemed optimistic because all patients in the endocrine-sensitive population had already been on prior therapy lines. Data with endocrine therapy from the later-line setting demonstrated a clinical benefit rate of 10–30%.

### CR1447 in the field of modern combination therapy with a CDK4/6 inhibitor

The current state-of-the-art treatment in a second-line setting comprises endocrine therapy along with a CDK4/6 inhibitor if it has not been applied in the first-line setting. In most clinical trials, the endocrine backbone has been fulvestrant, a modern selective estrogen receptor downregulator. Using this drug combination, an OR rate of 30–40%, a PFS of 9–16 months, and an OS of 30–40 months are likely to be expected (Turner *et al.* 2015, Sledge *et al.* 2017, Slamon *et al.* 2018). The benefits observed from the use of the single-agent CR1447 are far behind those reported from second-line phase III trial combinations. In our study, however, CR1447 exhibited signs of activity with a clinical benefit rate of 23.8% at week 24, a PFS of 5.1 months, and an OS of 24.6 months.

Data from older studies examining second-line endocrine therapies, such as AIs, medroxyprogesterone, or tamoxifen, have demonstrated comparable or better response rates than CR1447, with a longer response duration, PFS, and OS (Buzdar *et al.* 1997, Byrne *et al.* 1997, Russell *et al.* 1997, Bernhard *et al.* 1999, Kvinnsland *et al.* 2000). In particular, patients with a longer duration of first-line endocrine therapy experienced benefits when treated with CR1447, as illustrated in Fig. 1. Based on the available data, further development of CR1447 may prove useful considering endocrine-sensitive ER+ populations with low-volume disease. Another approach could be a combination trial with a modern CDK4/6 inhibitor, given that the drug combination may have minor overlapping toxicity.

In this study, the primary endpoint was also negative for (B), with no DC24, and a clinical DC12 of 12.5%. Patients with metastatic TNBC have an extremely poor prognosis (Leone *et al.* 2019). Only a few patients derive benefits from systemic treatment beyond second-line treatments (Schilling *et al.* 2019), with no approved targeted treatment in this setting. Atezolizumab, an anti-PDL-1 checkpoint inhibitor (Schmid *et al.* 2018), constitutes a new treatment approach for first-line therapy in PDL-1+ immune cells in TNBC. The use of atezolizumab plus chemotherapy was shown to be associated with a significantly better PFS than chemotherapy alone. For BRCA-mutated patients, olaparib, a poly(ADP-ribose) polymerase (PARP) inhibitor, was approved in later lines, exhibiting benefits against third-line chemotherapy regimens (Robson *et al.* 2017). Of note, not all patients with TNBC will derive benefits from these new approaches. However, in TNBC, AR+ is found in 10–45% of all cases (Sutton *et al.* 2012, Tang *et al.* 2012, Pistelli *et al.* 2014, Jongen *et al.* 2019). Such AR expression is generally associated with older patient age, lower KI-67, non-ductal histology (includes all other histologic subtypes including lobular, medullary, metaplastic, and apocrine), grading G1–2, and lower tumor-infiltrating lymphocyte levels (Dieci *et al.* 2019). In this trial, all patients with AR expression >0% were eligible in (B). Newer data from a trial that examined the role of a modern AR blocker (enzalutamide) demonstrated a better outcome for TNBC when AR expression was >10% (Traina *et al.* 2018). A subgroup analysis of this trial demonstrated a higher clinical benefit rate of 33% vs 25% if the AR expression was >10%. In this trial, the median PFS was 2.9 months in the IIT group vs 3.3 months for patients in the subgroup with AR expression >10%, demonstrating a poor outcome in metastatic TNBC.

In our trial, CR1447 failed to attain the primary endpoint for several different reasons, such as aggressive tumor type in the third-line setting and patient selection based on a low AR expression of >0%. Additionally, the accrual rate was low. For further CR1447 investigations in TNBC, only patients with an AR expression >10% should be included. The patients could also be selected based on the molecular subtype classification used by Lehmann *et al.* in which only luminal AR+ TNBC subtypes with a low KI-67 should be included (Lehmann *et al.* 2016). Of note, 5/8 patients in (B) displayed an AR expression rate ≤10%, which could partly explain the cohort’s poor outcome. Considering new treatments, including PARP inhibitors, immunotherapy, or drug conjugates (Caparica *et al.* 2019), CR1447 might be beneficial only in maintenance and combination settings in luminal AR+ TNBC subtypes.

Overall, CR1447 was well-tolerated and only grade 1–2 treatment-related toxicities were reported. The most common side effects were dry skin, breast pain, nausea, rash, and fatigue, along with elevated liver function and hypertriglyceridemia, which are typically associated with this drug (Zweifel *et al.* 2017). Patients with endocrine symptoms showed stable QoL scores during the treatment phase; however, disease progression occurred in most patients during follow-up. In the patients’ views, the CR1447 application was deemed acceptable, although approximately half of them would have preferred the use of tablets rather than an ointment. We can only speculate on these numbers. Maybe patients are more used to tablets intake.

## Conclusion

CR1447 is a new topical endocrine formulation, which has a simple everyday skin application. The expected DC24 was not met in either cohort (A/B). In (A) the DC24 was 23.8%, showing signs of efficacy in patients with endocrine-sensitive tumors. Nevertheless, the DC12 (secondary endpoint) was high with 61.9% in (A) (eligible population) and in (B) it was 12.5%. The drug was very well-tolerated, with few side effects and no treatment-related severe toxicities. Therefore, it might be an ideal candidate for further evaluation in a maintenance setting or when used in combination with other drugs.

Further investigations in endocrine-sensitive patients or in combination with a CDK4/6 inhibitor appear warranted. For further evaluation of CR1447 in TNBC, patient selection must be carefully based on the molecular subtype of TNBC and AR expression (>10%). Higher cut-off demonstrated better outcomes in TNBC. The drug might be evaluated in clearly AR+ (cut-off >10%) TNBC with a larger number of patients.

## Declaration of interest

W S is a scientific advisor for CURADIS. The other authors have nothing to disclose.

## Funding

This study did not receive any specific grant from any funding agency in the public, commercial, or not-for-profit sector.

## Author contribution statement

M V, W S, K M R, H H, Q L, K R, R v M, A M, B T: conceptualization, methodology, software; M V, Q L, M V, B T: data curation, writing - original draft preparation; M V, Q L, K M R, H H, A M, B T: visualization, investigation. B T: supervision; K M R, H H, Q L: software, validation; all: writing – reviewing and editing.
